# Clinical applications and future directions of Iodine-131-Metaiodobenzylguanidine therapy in neuroblastoma: from salvage treatment to frontline integration

**DOI:** 10.1007/s12149-025-02151-3

**Published:** 2026-01-13

**Authors:** Hoang Minh Chau Vu, Daiki Kayano, Hiroshi Wakabayashi, Rie Kuroda, Seigo Kinuya

**Affiliations:** 1 Division of Advanced Preventive Medical Science, Graduate School of Advanced Preventive Medical Sciences, Kanazawa University, Kanazawa University13-1 Takara- machi, Kanazawa, 920-8641 Ishikawa Japan; 2 Department of Nuclear Medicine, Kanazawa University Hospital, 13-1 Takara-machi, Kanazawa, 920-8641 Ishikawa Japan; 3 Department of Pediatrics, Kanazawa University Hospital, 13-1 Takara- machi, Kanazawa, 920-8641 Ishikawa Japan; 4 Department of Pediatrics, Ishikawa Prefectural Central Hospital, 2-1 Kuratsuki Higashi, Kanazawa City, 920-8530 Ishikawa Japan

**Keywords:** Neuroblastoma, ^131^I-MIBG, Immunotherapy

## Abstract

Neuroblastoma is the most common extracranial solid tumor in children and often requires complex, multimodal treatment. A major advancement in its treatment has been the development of metaiodobenzylguanidine (MIBG), a norepinephrine analog, for diagnostic imaging and targeted radiotherapy. This review provides a comprehensive overview of the clinical applications and recent advancements of ^131^I-MIBG in neuroblastoma treatment. The agent has exhibited substantial efficacy in newly diagnosed and relapsed or refractory disease, through strategies such as monotherapy, tandem dosing, and high-dose administration with stem cell transplantation support. Combination regimens incorporating radiosensitizers, chemotherapeutic agents, and novel targeted approaches-including histone deacetylase inhibitors and immunotherapy-have further broadened its therapeutic scope. Challenges remain, including nonavid tumors, radiation-related risks, and logistical constraints. However, with the recent regulatory approval of ^131^I-MIBG for neuroblastoma treatment in Japan, its role is expected to expand, paving the way for improved outcomes and more personalized, targeted approaches in pediatric oncology.

## Introduction

Neuroblastoma (NB) is an embryonal original tumor of the peripheral sympathetic nervous system and is the most common extracranial solid tumor in children, accounting for approximately 15% of pediatric cancer cases [1, 2]. Arising from neural crest cells, it most often develops in the adrenal glands or paraspinal ganglia [3]. Clinically, NB exhibits marked heterogeneity, with presentations ranging from spontaneous regression to highly aggressive metastatic disease in children [4, 5].

Diagnosis relies on a combination of imaging modalities—such as ultrasonography, computed tomography (CT), magnetic resonance imaging (MRI), and functional nuclear imaging—to determine disease extent [2]. Genetic profiling of tumors is equally important for risk stratification and identifying potential therapeutic targets [2, 6].

Risk classification guides treatment planning and incorporates factors including patient age, disease stage, histopathology, MYCN gene amplification, tumor cell ploidy, and segmental chromosomal abnormalities [7]. Patients are grouped into low-, intermediate-, and high-risk categories. Low-risk cases often require only observation or surgical resection, whereas intermediate-risk patients may need surgery combined with moderate chemotherapy [7–9]. High-risk cases demand intensive multimodal therapy, including induction chemotherapy, surgical resection, myeloablative chemotherapy with autologous stem cell transplantation, radiotherapy, nuclear medicine therapy, and immunotherapy [7, 10].

Prognosis varies considerably: survival exceeds 90% in low-risk patients but drops to approximately 50% at 5 years for high-risk patients despite aggressive management [4, 8].

This review examines the role of metaiodobenzylguanidine (MIBG) and recent advances in nuclear medicine for neuroblastoma therapy. A key milestone was the 2025 approval of ^131^I-MIBG by the Japanese Ministry of Health, Labour and Welfare, representing a major step forward for patient care.

## ^131^I-MIBG introduction

First synthesized in 1979, MIBG is a radiopharmaceutical agent widely used for diagnosing and treating neuroendocrine tumors, particularly neuroblastoma [11]. As a structural analog of norepinephrine, MIBG is actively transported into tumor cells via the norepinephrine transporter (NET) and stored in neurosecretory granules through vesicular monoamine transporters (VMAT) [12]. When labeled with the beta-emitting isotope iodine-131, ^131^I-MIBG delivers targeted radiation that induces DNA damage and tumor cell death, making it an effective therapeutic option [13].

After intravenous administration, ¹³¹I-MIBG is rapidly cleared from the circulation and shows a characteristic physiological biodistribution [12]. The tracer accumulates in organs with dense sympathetic innervation and high norepinephrine transporter (NET) expression, such as the salivary and lacrimal glands, thyroid, heart, and adrenal medulla, and is also seen in the liver, spleen, gastrointestinal tract, and urinary system, reflecting hepatic metabolism and renal and intestinal excretion [12, 13]. Thyroid blockade with stable iodine is routinely performed to prevent free radioiodine uptake and substantially reduces, though may not completely eliminate, thyroid visualization on MIBG scintigraphy. Approximately 30%–50% of the administered dose is excreted unchanged in urine within 1–2 days; hydration and frequent voiding help reduce bladder radiation exposure [14].

Therapeutic efficacy depends on factors affecting tumor MIBG uptake, including the density and function of NET on neuroblastoma cells, tumor burden and distribution, prior therapies that may modulate NET expression, and the delivered radiation dose, whereas MYCN amplification often reduces it. Other transporters, such as the plasma membrane monoamine transporter (PMAT) and VMAT, aid retention [15], multidrug resistance proteins (MRP1, MRP4) can promote efflux—an effect mitigated by inhibitors like probenecid [16]. Uptake may also improve with tumor differentiation, reduced tumor burden, favorable genetics, or transporter-inducing treatments, but can be impaired by certain medications [12, 17–19].

The specific activity of ^131^I-MIBG is a key determinant of therapeutic efficacy. Low-specific-activity (LSA) MIBG, produced via isotopic exchange, contains a significant proportion of nonradioactive (“cold”) MIBG, which competes with radioactive form of MIBG for NET uptake. This competition can lower tumor radiation delivery and increase off-target effects. In contrast, high-specific-activity (HSA) MIBG contains minimal cold MIBG, thereby enhancing uptake of the radioactive compound, delivering higher tumor doses, and improving therapeutic outcomes [20, 21]. However, it should be noted that the supply of HSA-MIBG in the United States has already been discontinued, which may limit its clinical availability and future research applications.

Although ^131^I-MIBG therapy is generally better tolerated than conventional chemotherapy or external beam radiation, it can still cause dose-limiting toxicities, most notably bone marrow suppression and radiation-related injury to certain organs. Proactive monitoring and supportive interventions are essential to reduce these risks. A summary of the major adverse effects and their management is provided in Table 1 [22–27].

**Table 1:** ^131^I-MIBG treatment side effects and management

Aspect	Early Side Effects	Late Side Effects	Management
Hematological toxicity	Thrombocytopenia, neutropenia, and anemia	Prolonged thrombocytopenia and neutropenia	**Prevention**: Prophylactic AHSCT **Treatment**: Transfusions, supportive care, and AHSCT if severe. **Follow-up**: Regular CBC
Thyroid toxicity	–	Hypothyroidism	**Prevention**: Thyroid blockage **Treatment**: Thyroid hormone replacement and monitoring thyroid function tests **Follow-up**: Lifelong thyroid function tests and thyroid cancer screening
Hepatic toxicity	Transient asymptomatic elevation of transaminases	Rare long-term hepatic dysfunction and persistent transient liver abnormalities	**Prevention**: liver function monitoring **Treatment**: Supportive care and specific interventions if severe **Follow-up**: liver function monitoring
Gastrointestinal toxicity	Nausea and vomiting shortly after treatment	–	Supportive care as needed
Secondary malignancies	–	Rare but reported cases	Regular monitoring during long-term follow-up (CBC, clinical examinations)

**Source**: references 22–27

**Abbreviations**: AHSCT, autologous hematopoietic stem cell transplantation; CBC, complete blood count

## Indications and contraindications

^131^I-MIBG therapy has become a vital option in the management of advanced neuroblastoma, particularly in relapsed or refractory cases. According to the European Association of Nuclear Medicine guidelines (2010), the National Comprehensive Cancer Network Clinical Practice Guideline, and the Japanese Society of Nuclear Medicine draft guidelines [28–30], the indications for ^131^I-MIBG therapy in neuroblastoma include the following:

- Stage III or IV neuroblastoma demonstrates MIBG uptake on the ^123^I-MIBG diagnostic scan as baseline. The term ‘MIBG uptake’ refers to visually appreciable accumulation of tracer in the primary tumor or metastatic lesions that are higher than the surrounding background and at least comparable to, or greater than, physiological liver uptake on planar images [12, 28–30]. Both planar scintigraphy and SPECT/CT are used in clinical practice. SPECT/CT is particularly useful for lesion localization, assessment of equivocal uptake, and dosimetric calculations, and is recommended whenever available in complex cases or when precise anatomical correlation is required.
- Typical high-risk or treatment-resistant neuroblastoma, including those who have relapsed or did not achieve remission with conventional therapy.
- Palliative care in inoperable or multifocal disease.

The key contraindications include:

- Non-avid disease: If a patient’s neuroblastoma does not show up on a diagnostic scan, ^131^I-MIBG therapy is contraindicated. In principle, absence of MIBG avidity on a properly performed diagnostic scan is considered a contraindication to ^131^I-MIBG therapy. However, false-negative studies can occasionally occur, for example, in patients with very small lesions, tumors with low or heterogeneous NET expressions, prior chemotherapy that has downregulated NET, or suboptimal imaging technique. In such situations, correlation with MRI and/or ¹⁸F-FDG PET/CT and, when appropriate, repeat MIBG imaging under optimized conditions should be considered before definitively classifying a tumor as MIBG-negative.
- Severe renal impairment: MIBG is renally excreted, and poor renal function can increase the whole-body radiation dose and toxicity.
- Minimal life expectancy (< 1–3 months): EANM cites < 3 months as a contraindication, whereas the Japanese guidelines use < 1 month. An exception can be made for refractory bone pain, where ^131^I-MIBG may still be administered as a compassionate palliative measure even in end-stage patients.
- Uncontrolled medical issues: Serious comorbidities or risk factors that could be exacerbated by therapy or require strict isolation. Additionally, urinary incontinence can complicate the required radiation safety isolation, as ^131^I is excreted in the urine, potentially leading to contamination.
- Pregnancy would be a contraindication in older patients because of the radiation risk to the fetus, although this is rarely relevant in pediatric neuroblastoma.

## ^131^I-MIBG in neuroblastoma treatment

When radiolabeled with ^131^I, MIBG enables targeted radionuclide therapy for neuroblastoma, providing a systemic means of delivering radiation to tumor sites. ^131^I-MIBG was first introduced as a therapeutic option in the 1980s, with Treuner et al. reporting the earliest clinical use in 1986 [31].

Since then, numerous phase I and II trials have investigated ^131^I-MIBG monotherapy in relapsed or refractory neuroblastoma. These studies have demonstrated that ^131^I-MIBG can achieve objective responses even in chemotherapy-resistant disease, although reported rates vary widely from 0% to 75% [32]. A 2022 meta-analysis of 26 clinical trials involving 883 patients estimated a pooled objective response rate of approximately 39% [33]. Responses ranged from complete or partial tumor regression to durable disease stabilization in select cases. Considering its efficacy and absence of cross-resistance with conventional cytotoxic drugs, ^131^I-MIBG has become an important salvage therapy and is now available in specialized centers worldwide. However, considerable variation in treatment timing, eligibility criteria, and clinical application persists, underscoring the need for clearer definitions of therapeutic intent and target patient populations to ensure consistency and reproducibility of results.

## ^131^I-MIBG monotherapy

### Conventional ^131^I-MIBG monotherapy for relapsed or refractory neuroblastoma

Conventional ^131^I-MIBG monotherapy has shown consistent activity in patients with relapsed or chemotherapy-refractory neuroblastoma, providing a vital option for patients with few alternatives. Evidence from early studies in the 1980s and 1990s, as well as more recent trials, supports its efficacy as a single agent, with key representative findings summarized below.

Across these studies, ^131^I-MIBG achieved objective responses in approximately one-third of patients with refractory neuroblastoma. In a 1991 German multicenter trial, Klingebiel et al. reported a 36% objective response rate (ORR) among 47 children [34]. Similarly, a 1992 UK Phase I/II study by Lashford et al. noted partial remissions in heavily pretreated patients, with responses lasting 4–9 months [35]. Garaventa et al. (1999) evaluated 43 patients with residual disease post–first-line therapy and achieved a 37% ORR (1 complete response [CR], 10 partial responses [PR]) among 30 stage 4 cases, with ~ 50% maintaining stable disease (SD) [22]. This study used up to five serial ^131^I-MIBG infusions (median 3), anticipating later multi-infusion strategies. In 2007, a US phase II study by Matthay et al. involving 164 patients confirmed a 36% ORR (12% CR), reinforcing ^131^I-MIBG’s robust single-agent activity across diverse sites and treatment histories [36].

Beyond inducing objective responses, ^131^I-MIBG frequently provides meaningful disease stabilization. In Matthay’s 2007 study, approximately 40% of patients achieved SD lasting beyond 12 weeks, offering symptom relief and serving as a bridge to subsequent therapies [36]. However, without further treatment, most patients with responses or SD eventually progress. The median progression-free survival (PFS) typically ranges from 6 to 12 months in responders but is shorter (< 6 months) in non-responders. In a 2020 retrospective series, Rubio et al. reported a median PFS of 7 months overall—15 months for responders versus 1 month for non-responders [37]. Progression often occurs within 3 months in > 85% of non-responders, whereas responders rarely progress before 6 months, with some experiencing remissions exceeding 2 years.

Although long-term cures with ^131^I-MIBG monotherapy are uncommon in relapsed neuroblastoma, integration into salvage regimens can extend survival. Reported 5-year overall survival (OS) rates range from 20% to 35%, largely dependent on subsequent treatments. In Garaventa’s Italian cohort, 40% of stage 4 patients were alive at a median follow-up of 5 years from diagnosis, underscoring ^131^I-MIBG’s value in improving outcomes when used in combination strategies [22]. Major studies of conventional ^131^I-MIBG monotherapy in relapsed/refractory neuroblastoma are summarized in Table 2 [22, 31, 33–36, 38].

**Table 2:** Select studies of conventional ^131^i-mibg monotherapy in relapsed/refractory neuroblastoma

Study (Year)	Population	^131^I-MIBG Dose	Key Efficacy Outcomes	Notable Safety Outcomes
Treuner et al. (1986) [31]	9 children, relapsed NB (phase I)	1–3 infusions; ~3700–5550 MBq per course (fixed dose)	2 PR (22%); 4 SD; transient pain relief in some cases	Marrow suppression dose-dependent; no serious non-hematological toxicity reported (small sample)
Klingebiel et al. (1991) [34]	47 pts, refractory NB (multi-center)	1–2 infusions; max 444 MBq/kg	ORR 36% (8% CR, ~ 28% PR); SD in additional ~ 30%; median response ~ 6 mo	~ 80% Grade 3–4 neutropenia; ~70% thrombocytopenia; no treatment-related mortality reported
Lashford et al. (1992) [35]	25 pts, chemoresistant NB (UKCCSG)	1–3 infusions; dose escalated up to 444 MBq/kg	6 major responses (2 CR) lasting 4–9 mo; 2 minor responses (> 12 mo)	Dose-limiting hematological toxicity at 444 MBq/kg; transient hypertension in a few during infusion; manageable with support
Garaventa et al. (1999) [22]	43 pts (13 stage 3, 30 stage 4) with residual disease after frontline therapy	1–5 courses; 2516–5513 MBq per course (~ 74–259 MBq/kg)	Stage 4: ORR 37% (1 CR, 10 PR); Stage 3: ORR 15% (2 PR); overall clinical benefit (CR + PR + SD) ~ 70%	100% hematological toxicity (some required stem cells after ≥ 3 courses); 2 cases secondary leukemia (5%); 1 fatal interstitial pneumonitis; 49% with reduced thyroid function
Matthay et al. (2007) [36]	164 pts, refractory NB (multi-institution)	1 infusion; measured dosimetry to ~ 444 MBq/kg on average	ORR 36% (12% CR, 24% PR); 49% SD; 15% PD as the best response. Higher ORR in older patients and those with fewer prior regimens. 1-year OS 59%.	85% Grade 3–4 neutropenia, 82% thrombocytopenia; ~10% prolonged cytopenia > 6 weeks; <5% severe non-hematological toxicity (no grade 4 organ toxicities). Thyroid dysfunction ~ 30% at 2 yrs (subclinical in most).
Polishchuk et al. (2011**)** [38]	32 pts (children, adolescents, adults) relapsed NB	1 infusion; median ~ 444 MBq/kg (range 296–666)	ORR 41% in < 12 yrs vs. 33% in ≥ 12 yrs (no significant difference); median PFS ~ 6 mo; symptomatic improvement in 70%.	Similar chematological toxicity across ages; no age-related increase in organ toxicity. A total of 50% developed hypothyroidism over the long-term follow-up.
He et al. (2022) (Meta-analysis) [33]	Pooled 883 pts from 26 trials (1985–2021)	Monotherapy arms (various doses)	Pooled ORR 39% (95% CI ~ 34–44%); SD 31%; PD ~ 22%. Pooled 1-year OS 64%, 5-year OS 32% for MIBG monotherapy.	Pooled ≥ Grade 3 hematological toxicity: thrombocytopenia 53%, neutropenia 58%. Higher hematologic toxicity in combination regimens. No significant acute non-hematological toxicity in the pooled analysis.

### Tandem ^131^I-MIBG therapy

Conventional ^131^I-MIBG therapy for relapsed neuroblastoma is often administered as a single infusion. In contrast, tandem ^131^I-MIBG therapy delivers two closely timed infusions to enhance treatment efficacy in relapsed or refractory disease. While single-dose therapy frequently yields PR or SD, the tandem approach aims to deepen tumor responses. The second infusion is typically administered within weeks of the first, often with hematopoietic stem cell support if hematological parameters permit. In a 2011 University of Pennsylvania study, 76 children received an initial infusion of 666 MBq/kg [39]. Of these, 30% responded (5% CR, 25% PR) and 49% achieved SD. Among the 41 patients eligible for a second infusion within 6–12 weeks, 29% experienced additional tumor reduction, resulting in an overall tumor burden decrease in 39% of the cohort. These findings indicate that tandem ^131^I-MIBG therapy is both feasible and capable of improving disease control in patients who achieve at least SD after the initial dose. Evidence for tandem ^131^I-MIBG infusions is presented in Table 3 [39–41].

**Table 3:** Studies of tandem ^131^I-MIBG infusions for neuroblastoma

Study (Year)	Population	Tandem Regimen	Efficacy Outcomes	Safety Outcomes
Matthay et al. (2009)​ [40]	21 patients, high-risk NB	2 infusions, 14 days apart; ASCR 2 weeks after the second	2 PR, 8MR, 3 SD, and 7 PD	**No dose-limiting toxicities were** observed. Grade 3 non-hematological toxicity occurred in six patients.
Johnson et al. (2011) [39]	76 patients, relapsed NB after ≥ 1 prior regimen	1st 666 MBq/kg; if ≥ SD, 2nd 666 MBq/kg, second dose administered within 100 days of the first (median interval: 59 days); PBSC given after 2nd	**First infusion**: ORR = 30% (CR 6.6%, PR 23.7%) **Second infusion (41 patients)**: ORR = 29% (CR 12.2%, PR 17.1%) **Overall (2 infusions)**: 39% had disease reduction (PR/CR to one infusion, SD or better to the other)	-Hematological toxicity was primary: 85% required stem cell support after 2nd infusion 80% needed platelet transfusions after 1st infusion. -Non-hematological toxicities were rare (e.g., fever with neutropenia in ~ 12%) -No failure in neutrophil recovery
Altini et al. (2022) [41]	13 patients, relapsed NB	Tandem: two infusions with a dosimetry approach	1 PD, 4 PR, plus 8 SD.	All cases suffer from myelosuppression and need blood transfusions.

**Abbreviations**: NB, neuroblastoma; ORR, objective response rate (CR + PR); CR, complete response; PR, partial response; SD, stable disease; PD, progressive disease; OS, overall survival

### Submyeloablative ^131^I-MIBG doses and outpatient therapy

Submyeloablative ^131^I-MIBG therapy administers low to moderate doses (typically 37–444 MBq/kg) to control tumor growth or alleviate symptoms in advanced neuroblastoma while minimizing treatment-related toxicity. Unlike high-dose regimens, this approach permits bone marrow recovery without stem cell support, reducing the risk of severe cytopenia and avoiding prolonged hospitalization.

For example, Vishnu et al. (2024, India) treated 39 children with doses of 37–74 MBq/kg, achieving disease regression in 18 patients and significant pain relief in many, with only mild nausea and minimal hematological issues. This strategy provides a safe, resource-efficient option for palliative care or disease control when high-dose therapy is not feasible [42]. Submyeloablative ^131^I-MIBG regimens are outlined in Table 4 [22, 37, 42].

**Table 4:** Submyeloablative ^131^I-MIBG therapy approaches

Approach/Study	Dose Strategy	Clinical context and outcomes	Advantages	Limitations
Ultra-low dose (Vishnu 2024) [42]	37–74 MBq/kg; often single infusion	Resource-limited setting, palliative intent on 39 children. Outcomes: 46% had disease regression on the MIBG scan; notable pain relief in many.	Minimal toxicity (no ≥ Grade 3 marrow toxicity); can be outpatient; cheap.	Low ORR (~ 10–20% PR); mainly symptomatic benefit; not curative.
Moderate repeated doses (Garaventa 1999) [22]	~ 148 MBq/kg per course, repeated every ~ 8 weeks (median 3 courses)	Residual disease post-chemotherapy. Outcomes: ORR 30–40% over multiple courses; some converted PR→CR with successive doses.	Avoids stem cell transplant; cumulative dosing possible; can achieve significant tumor reduction over time.	Requires careful count recovery between cycles; risk of cumulative myelosuppression and secondary leukemia (observed ~ 5%).
Fractionated dosimetry (Rubio 2020) [37]	444 MBq/kg, then second dose 2 weeks later, adjusted to a whole-body 4 Gy total	Mixed refractory/relapse. Outcomes: ORR 47%; the fractionated group had a longer median PFS (not significant). Allowed ~ 666 MBq/kg total in 2 steps with less toxicity than one 666 MBq/kg dose.	Optimizes tumor dose while controlling normal organ dose; somewhat mimics tandem without full myeloablation; improved tolerability.	

**Abbreviations**: NB, neuroblastoma; ORR, objective response rate (CR + PR); CR, complete response; PR, partial response; SD, stable disease; PD, progressive disease; OS, overall survival

### ^131^I-MIBG monotherapy in a newly diagnosed neuroblastoma

^131^I-MIBG therapy in newly diagnosed high-risk neuroblastoma represents a rational strategy to achieve early cytoreduction by directly targeting norepinephrine transporter–expressing tumour cells at the outset of treatment. In a phase II trial from the Netherlands, de Kraker et al. administered two upfront cycles of ^131^I-MIBG (7.4 GBq followed by 3.7 GBq) before conventional chemotherapy in 44 children with stage 4 neuroblastoma over 1 year of age [43]. The overall response rate (ORR) after ^131^I-MIBG alone was 66%, with substantial tumour shrinkage in approximately two-thirds of patients, thereby facilitating subsequent surgical resection in selected cases.

After completion of the full multimodality regimen—including chemotherapy, surgery where indicated, and further consolidative treatment—the ORR increased to 73% [43]. These findings suggest that integrating ^131^I-MIBG at diagnosis can deepen initial responses and may improve operability of bulky disease. However, this approach also introduces practical challenges, such as the need for close coordination between nuclear medicine and oncology teams, age-appropriate isolation facilities, and meticulous toxicity monitoring in very young children. Overall, available data support the feasibility and potential benefit of ^131^I-MIBG as a first-line component of therapy in carefully selected high-risk patients, while underscoring the need for further prospective trials to define its optimal timing and dosing schedule.

## High-dose ^131^I-MIBG monotherapy (myeloablative regimens) with stem cell transplantation

High-dose ^131^I-MIBG monotherapy followed by myeloablative chemotherapy and haematopoietic stem cell transplantation (HSCT) aims to maximise tumour-targeted radiation while safely managing the profound marrow toxicity associated with dose escalation. Conventional ^131^I-MIBG therapy is usually limited to approximately 444 MBq/kg by bone marrow tolerance; escalation to around 666 MBq/kg enables higher tumour doses but predictably induces marrow ablation, necessitating autologous or allogeneic stem cell rescue.

In a Japanese phase I/II study, Kuroda et al. evaluated this strategy in eight patients with high-risk neuroblastoma, six newly diagnosed and two with relapsed disease, who received 666 MBq/kg of ^131^I-MIBG followed by a single myeloablative chemotherapy course and autologous stem cell transplantation (ASCT) [44]. High-dose ^131^I-MIBG was delivered without dose-limiting toxicity; the anticipated myelosuppression did not delay conditioning or transplantation, and no unexpected grade 4 non-haematologic adverse events were reported. In two relapsed patients, the regimen was combined with killer-cell immunoglobulin-like receptor ligand–mismatched cord blood transplantation to exploit a potential graft-versus-tumour effect, which proved feasible and well tolerated. These data indicate that, with appropriate stem cell support, high-dose ^131^I-MIBG can safely replace or complement conventional chemotherapy in the myeloablative phase, particularly in the setting of minimal residual disease.

The Children’s Oncology Group (COG) evaluated integration of ^131^I-MIBG into the conditioning phase before busulfan/melphalan (Bu/Mel)–ASCT, the standard consolidation for high-risk neuroblastoma. In the pilot trial by Weiss et al., 68 newly diagnosed high-risk patients received induction chemotherapy followed by ^131^I-MIBG at 444, 555, or 666 MBq/kg approximately 1 month before Bu/Mel-ASCT [45]. The approach was feasible: 86% of patients completing induction proceeded to ^131^I-MIBG, and 82% subsequently underwent transplant. However, sinusoidal obstruction syndrome (SOS) occurred in about 25% of patients at the higher ^131^I-MIBG doses, prompting a protocol amendment that recommended 555 MBq/kg as the preferred activity for future studies. At this dose, ^131^I-MIBG delivery remained highly feasible (96%), and 81% of patients completed Bu/Mel-ASCT, although one treatment-related death from SOS highlighted the need for careful hepatic monitoring when combining intensive radiopharmaceutical therapy with hepatotoxic conditioning.

Kayano et al. subsequently reported a Japanese cohort of 19 patients with refractory or relapsed high-risk neuroblastoma (median age 7.9 years) treated with a single ^131^I-MIBG dose of 444–666 MBq/kg [46]. The regimen yielded three complete and two partial responses, but was associated with substantial haematologic toxicity: all patients experienced grade 3–4 neutropenia and thrombocytopenia, and 53% developed grade 3–4 anaemia. Stem cell rescue was implemented in 17 patients (5 autologous and 12 allogeneic), underscoring the need for planned HSCT support when pursuing this level of dose escalation.

Collectively, current evidence supports high-dose ^131^I-MIBG with HSCT as a promising intensification strategy that can increase tumour radiation dosing without substantially delaying definitive consolidation. Remaining challenges include the risk of SOS, the logistics of coordinating radiopharmaceutical administration and transplant timing, and the lack of definitive survival data. The ongoing COG ANBL1531 phase III trial, which randomizes patients to induction chemotherapy with or without ^131^I-MIBG before ASCT, is expected to clarify the true incremental benefit of this approach. Key clinical studies combining ^131^I-MIBG with stem cell transplantation are summarized in Table 5 [44–46].

**Table 5:** Studies of ^131^I-MIBG combined with stem cell transplantation in high-risk neuroblastoma

Study (Year)	Design	Population	MIBG Regimen	Outcomes	Safety
Kuroda et al. (2022) [44] Upfront high-dose MIBG + SCT	MIBG as part of upfront consolidation (pre-transplant)	6 pts, newly diagnosed high-risk (Japan)	1 MIBG at 666 MBq/kg before single-course HDCT + SCT	All 6 reached transplant in at least SD. 4 had MIBG-CR on the scan​ . As of the report, all are alive at a median of 18 months (*n* = 6, small sample).	Shows intensive upfront MIBG is tolerable in new pts. Long-term benefit TBD
Weiss et al. (2021) [45] – COG Pilot Induction MIBG	MIBG during induction, after 2–4 chemo cycles	68 pts, newly dx high-risk (COG A3973 backbone)	One MIBG infusion during induction (dose 444–666 MBq/kg per escalation); then standard Bu/Mel transplant	Feasibility met: 87% got MIBG per protocol​ End-induction ORR 71.7% (vs. ~ 70% in non-MIBG trials​). 19% progressed before transplant​ 2-year OS not yet reported (pilot not powered for efficacy).	Initial design caused 2 SOS cases; after ≥ 10 weeks, no SOS. MIBG added ~ 1 month to the induction length. Demonstrated logistic feasibility across multiple centers. Now being tested in phase III.
Kayano et al. (2020**)** [46] – High-dose MIBG + SCT in relapsed/refractory	High-dose MIBG in refractory or relapsed high-risk neuroblastoma (pre-transplant)	19pts, relapsed or refractory high-risk (Japan)	Single high-dose ¹³¹I-MIBG 444–666 MBq/kg IV, 17/19 received SCT after MIBG (5 autologous, 12 allogeneic).	Overall initial response rate 26% (CR 3, PR 2; SD 10, PD 3, NE 1). EFS after MIBG: 42% at 1 year, 16% at 5 years; OS after MIBG: 58% at 1 year, 42% at 5 years.	Grade 3–4 hematologic toxicity is common. on-hematologic AEs mostly grade 1–2. Only one patient had grade 3 anorexia/nausea; no other severe non-hematologic toxicities were reported.

**Abbreviations**: SIOPEN, Société Internationale d’Oncologie Pédiatrique Europe Neuroblastoma; COG, Children’s Oncology Group; Bu/Mel, Busulfan + Melphalan high-dose chemo; HDCT, high-dose chemotherapy; SCT, stem cell transplant; SOS, sinusoidal obstruction syndrome; EFS, event-free survival; OS, overall survival

## ^131^I-MIBG combination with chemotherapy and with/without stem cell transplantation

Efforts to enhance the efficacy of ^131^I-MIBG in relapsed or refractory neuroblastoma have focused on combining it with radiosensitising chemotherapies and high-dose regimens supported by HSCT. These strategies aim either to increase the depth of response in heavily pretreated patients or to render more patients eligible for consolidative transplantation.

### Combination with radiosensitising chemotherapy

Topotecan, a topoisomerase I inhibitor, demonstrated radiosensitising potential in preclinical neuroblastoma models and was subsequently incorporated into early clinical studies. Pilot work established the feasibility of topotecan combined with ^131^I-MIBG in children, with myelosuppression as the principal dose-limiting toxicity. Building on this, the New Approaches to Neuroblastoma Therapy (NANT) consortium conducted a phase I trial of ^131^I-MIBG with vincristine and irinotecan. This study showed that 666 MBq/kg of ^131^I-MIBG could be safely administered with vincristine and a shortened 5-day irinotecan schedule, which reduced the incidence of severe diarrhea compared with longer irinotecan regimens [47]. A subsequent phase I/II trial using this combination in 32 patients achieved a 28% response rate, confirming both feasibility and clinically meaningful activity at the 666 MBq/kg dose level.

To explore novel radiosensitizers, NANT also evaluated vorinostat, a histone deacetylase inhibitor, combined with ^131^I-MIBG. In a phase I study, 666 MBq/kg of ^131^I-MIBG plus vorinostat 180 mg/m² was tolerable and produced a 17% response rate; higher vorinostat doses led to excess haematologic toxicity, particularly thrombocytopenia [48]. A later randomized phase II NANT trial compared three regimens: ^131^I-MIBG alone, ^131^I-MIBG plus vincristine/irinotecan, and ^131^I-MIBG plus vorinostat [49]. Response rates were 14% for ^131^I-MIBG alone, 14% for the vincristine/irinotecan arm (with greater toxicity), and 32% for the vorinostat combination, which demonstrated the most favourable balance between efficacy and tolerability. Vorinostat, therefore, emerged as the preferred chemotherapy partner for further investigation in this setting.

### Combination with High-dose chemotherapy and stem cell transplantation

^131^I-MIBG has also been integrated with high-dose chemotherapy and ASCT in relapsed or refractory neuroblastoma. A NANT phase II study combined ^131^I-MIBG (296–444 MBq/kg, adjusted for renal function) with carboplatin, etoposide, and melphalan, followed by ASCT [50]. Among 41 patients with refractory or progressive disease, the overall response rate was 10%; however, in a small subgroup who had already achieved partial remission prior to enrolment, the response rate reached 38%, suggesting that this regimen may be particularly beneficial as consolidation after cytoreduction. Veno-occlusive liver disease occurred in 12% of patients and was dose-limiting in 10% (5/50), underscoring the importance of careful hepatic risk assessment and dose planning.

In Europe, the French MIITOP phase II trial (2008–2015) investigated tandem ^131^I-MIBG infusions combined with 5 days of topotecan, followed by ASCT in children with refractory or relapsed disease [51]. The observed response rate was modest (13%), but a substantial proportion of patients achieved stable disease, allowing them to proceed to consolidative busulfan/melphalan transplantation. Some of these patients achieved long-term survival with acceptable acute toxicity profiles. These findings suggest that, even when objective responses are limited, ^131^I-MIBG–based combination regimens can contribute to disease stabilisation and facilitate potentially curative HSCT.

Taken together, clinical data indicate that combining ^131^I-MIBG with radiosensitising chemotherapies, particularly vorinostat or topotecan, is safe and can improve response rates or achieve durable disease stabilisation in relapsed or refractory neuroblastoma. Regimens that incorporate ^131^I-MIBG into intensive chemotherapy and ASCT appear especially useful as consolidation in patients with chemosensitive disease. Ongoing and future trials will be crucial for refining patient selection, optimising dosing, and determining the comparative benefits of competing combination strategies. Key clinical studies combining ^131^I-MIBG with chemotherapy in relapsed/refractory neuroblastoma are summarized in Table 6 [47–52].

**Table 6:** Key clinical studies of ^131^I-MIBG combined with chemotherapy in relapsed/refractory neuroblastoma

Study (Year)	Population	MIBG Dose	Chemotherapy Regimen	Efficacy Outcomes	Safety/Adverse Events
Matthay et al. JCO 2006 (NANT Phase I) [52]	Relapsed/refractory; *n* = 24	444 MBq/kg (MTD)	CEM (carboplatin, etoposide, melphalan) before ASCT	MTD 444 MBq/kg with ASCT support; preliminary responses seen (no formal ORR reported)	Dose-limiting myelosuppression mitigated by stem cells; non-hematological toxicity minimal
DuBois et al. BJC 2015 (Phase I/II) [47]	Relapsed/refractory; *n* = 32 (1–30 y)	666 MBq/kg	Vincristine (Day 0) + Irinotecan (Days 0–4), 5-day course	ORR 28% (5 CR + 4 PR); responses in 9/32 patients	No DLTs; common toxicities: neutropenia, thrombocytopenia, diarrhea (grade 3 in. 6%); less diarrhea against protracted irinotecan
DuBois et al. CCR 2015 (Phase I) [48]	Relapsed/refractory; *n* = 27	444–666 MBq/kg (escalating)	Vorinostat (HDAC inhibitor) orally ×14 days	Recommended phase II dose: 666 MBq/kg MIBG + 180 mg/m² vorinostat; ORR 12% overall (17% at RP2D)	DLTs at higher vorinostat dose (grade 4 hypokalemia, bleeding); mostly hematological toxicity (neutropenia, thrombocytopenia)
DuBois et al. JCO 2021 (NANT Randomized Phase II) [49]	Relapsed/refractory; *n* = 105, 3 arms	666 MBq/kg (all arms)	Vincristine + Irinotecan; Arm C: Vorinostat	ORR after 1 cycle: 14% (Arm A), 14% (Arm B), 32% (Arm C). Best ORR after 2 cycles: 14%, 17%, 35% (A, B, C). Arm C selected as the superior regimen (not statistically compared)	Arm A: 19% ≥grade3 non-hematological toxicity; Arm B: 49% (↑GI toxicity); Arm C: 35%. Hematological toxicities in all (requiring ASCT support in the trial design). No unexpected organ toxicities were reported.
Yanik et al. BBMT 2015 [50]	Primary refractory/progressive (*n* = 42) and PR after induction (*n* = 8)	296 or 444 MBq/kg (by GFR strata)	CEM high-dose chemo + ASCT (myeloablative therapy)	ORR 10% in the refractory/progressive cohort; ORR 38% in the PR cohort. 3-year OS 62% (refractory) and 75% (PR); 3-year EFS ~ 20% and 38%, respectively	Transient hepatic SOS is 10% (5/50 dose-limiting); median engraftment times are 10 days for neutrophils and 15 days for platelets. Other non-hematological DLTs in 6/50 (including SOS). Manageable with supportive care.
Sevrin et al. PBC 2023(MIITOP Phase II – France) [51]	Relapsed or very-high-risk refractory; *n* = 30	~ 2 × 4-Gy WB dose (2 MIBG infusions)	Topotecan (Days 1–5 and 21–25) + ASCT; optional Bu/Mel transplant after	ORR 13% overall (19% in the induction-poor-response subset); 2-year EFS 17%. Disease control (≥ SD) in 81% of patients without prior transplant; enabled 11 patients to proceed to Bu/Mel, with 4 long-term survivors.	Acute non-hematological toxicity minimal (no grade ≥ 3; only grade 2 nausea/vomiting in some). Hematological toxicity was universal (all patients had ASCT). Post-MIBG liver toxicity low; Bu/Mel-related toxicity not worsened by prior MIBG

**Abbreviations**: CR, complete response; PR, partial response; ORR, overall response rate (CR + PR); SD, stable disease; DLT, dose-limiting toxicity; HDAC, histone deacetylase; RP2D, recommended phase 2 dose; GI, gastrointestinal; CEM, carboplatin, etoposide, melphalan; ASCT, autologous stem cell transplant; SOS, sinusoidal obstructive syndrome (veno-occlusive disease); WB, whole body; Bu/Mel, busulfan, melphalan

### Incorporating ¹³¹I-MIBG into frontline (Newly Diagnosed) therapy

Building on the success of ^131^I-MIBG in refractory neuroblastoma, several groups have explored introducing ^131^I-MIBG earlier in the treatment course for newly diagnosed high-risk patients. The principal objectives are to augment initial tumour shrinkage, more effectively clear metastatic disease, and thereby improve long-term remission rates. Two complementary strategies have emerged: (1) incorporating ^131^I-MIBG into induction chemotherapy for patients with extensive metastatic burden, and (2) using ^131^I-MIBG as part of high-dose consolidation regimens.

An Italian pilot study by Mastrangelo et al. evaluated ^131^I-MIBG during rapid-induction chemotherapy in 13 children with newly diagnosed stage 4 neuroblastoma [53]. In this protocol, ^131^I-MIBG (up to 614 MBq/kg) was administered on day 10 of an intensive 5-drug chemotherapy cycle (cisplatin, cyclophosphamide, vincristine, etoposide, and doxorubicin). The only significant toxicity attributable to ^131^I-MIBG was haematologic, and its severity was comparable to that expected from chemotherapy alone, even in patients with extensive bone marrow involvement. By 40 days from treatment initiation, two patients achieved a complete response (CR), six very good partial response (VGPR), and four partial response (PR), yielding ≥ PR in 12 of 13 patients. Notably, the deepest responses (CR/VGPR) were observed in those who received higher ^131^I-MIBG doses (~ 592 MBq/kg), supporting a dose–response relationship.

These pilot data show that adding ^131^I-MIBG to upfront multi-agent chemotherapy can markedly increase early response rates without introducing prohibitive non-haematologic toxicity. Nevertheless, wider implementation requires careful logistical planning, including coordination of nuclear medicine facilities, radiation safety precautions during induction, and integration with subsequent surgery and HSCT. Prospective, randomized trials are needed to determine whether such intensified frontline regimens translate into superior event-free and overall survival compared with current standard high-risk protocols.

## ^131^I-MIBG combination with other therapies

Hyperbaric oxygen therapy (HBOT) has been investigated as a means to enhance ^131^I-MIBG therapy for neuroblastoma by mitigating tumor hypoxia, a factor that diminishes radiation sensitivity. In a 1995 pilot study, Voûte et al. treated 27 children with recurrent stage 4 neuroblastoma using ^131^I-MIBG (7400 MBq initially, then 3700 MBq) followed by 4–5 daily HBOT sessions at 3 atmospheres for 75 min [54]. The 28-month survival rate was 32% compared with 12% among 36 patients who received MIBG alone, suggesting that HBOT can potentiate radiation-induced DNA damage without adding toxicity. Nonetheless, the absence of large confirmatory trials has restricted its application to specialized centers.

Another approach paired ^131^I-MIBG with arsenic trioxide (ATO) to amplify radiation-induced cell death. In a phase II study by Modak et al., 19 neuroblastoma patients received ^131^I-MIBG (444 or 666 MBq/kg) plus 10 days of ATO [55]. No patient achieved an objective response, with seven showing PD and 12 showing SD/minimal disease. Toxicity matched ^131^I-MIBG alone, primarily myelosuppression; however, ATO provided no clear benefit, halting its further use in this context.

Emerging strategies include combining ^131^I-MIBG with dinutuximab (anti-GD2 immunotherapy) in an ongoing phase I trial (NANT 2017-01), investigating whether radiation can augment immunotherapy [56]. Early findings support feasibility; however, efficacy results are pending. Case reports also suggest potential synergy between ^131^I-MIBG and Poly ADP-ribose)polymerase-1 (PARP) inhibitors such as talazoparib, particularly in patients harboring DNA repair defects. For example, one patient with PALB2 mutations achieved nine months of stable disease. Additional combinations—such as ^131^I-MIBG with ALK inhibitors or immune checkpoint blockade—are under investigation, though clinical data remain forthcoming.

## Treatment response and prognosis with the 123 I-MIBG scan

Since the 1980s, ^123^I-MIBG scintigraphy has been a key tool in the management of neuroblastoma, one of the most common pediatric cancers. Initially adopted for the detection of bone and soft tissue lesions, it demonstrated superior sensitivity and specificity compared with traditional bone scintigraphy [57, 58]. By the 1990s, ^123^I-MIBG had become an essential component of neuroblastoma staging protocols, serving as a complement to CT/MRI imaging and bone marrow evaluations. After that, ¹²³I-MIBG scintigraphy becomes the standard imaging modality for diagnosis, staging, and response assessment in MIBG-avid neuroblastoma. Because ¹²³I- and ¹³¹I-MIBG share the same molecular structure and are taken up via the norepinephrine transporter, diagnostic ¹²³I-MIBG imaging serves as a surrogate for predicting tumor avidity for therapeutic ¹³¹I-MIBG. In this therapy-focused review, ¹²³I-MIBG scans are therefore discussed only in terms of their role in selecting candidates for ¹³¹I-MIBG therapy, providing baseline semi-quantitative scores to guide treatment dosimetry, and evaluating response after ¹³¹I-MIBG administration. Over time, its role expanded beyond diagnosis to include prognostic assessment, with the development of semiquantitative scoring systems—such as the Curie and SIOPEN scores—in the 2000s to objectively quantify disease burden. Standardized through international initiatives like the 2010 International Neuroblastoma Risk Group Task Force, these tools have become invaluable for evaluating treatment response and predicting patient outcomes [59]. It should be noted that approximately 10% of neuroblastomas are MIBG-negative or show only minimal tracer accumulation [37]. In such patients, ¹²³I-MIBG scintigraphy is not suitable for response assessment or dosimetry, and alternative imaging modalities such as ¹⁸F-FDG PET/CT and MRI are preferred. These tumors are also not candidates for ¹³¹I-MIBG therapy and require other systemic treatment approaches.

^123^I-MIBG scintigraphy plays a pivotal role in assessing treatment response, particularly following induction chemotherapy, and provides powerful prognostic information. In a study by Yanik et al. involving 237 high-risk neuroblastoma patients, those with a Curie score > 2 after induction had a 3-year event-free survival (EFS) of roughly 15%, compared with ~ 45% for patients scoring ≤ 2 [59]. Similarly, the SIOPEN group identified a post-induction SIOPEN score > 3 as marking “ultra–high-risk” disease, with a 5-year EFS of about 16% versus ~ 43% for scores ≤ 3 [60]. A score of 0—indicating complete clearance of ^123^I-MIBG-avid skeletal lesions—correlated with the most favorable outcomes. Conversely, persistent ^123^I-MIBG uptake after induction signals a heightened risk of relapse and may prompt treatment intensification or alternative strategies.

Serial ^123^I-MIBG scans are used throughout the treatment to monitor the response and detect residual or recurrent disease. The International Neuroblastoma Response Criteria (INRC) integrate ^123^I-MIBG findings into metastatic response assessment, requiring complete disappearance of all ^123^I-MIBG-avid lesions to declare a complete metastatic response [61]. Scans are typically obtained at the end of induction to inform consolidation planning—often before stem cell transplantation—and again at the end of therapy to confirm remission. Residual uptake at this stage frequently leads to site-directed interventions, such as targeted radiation.

During follow-up, ^123^I-MIBG scans help detect recurrent disease, particularly in high-risk cases, either through scheduled surveillance or when prompted by symptoms or rising tumor markers. In relapsed or refractory cases, ^123^I-MIBG scans are essential for restaging and determining eligibility for ^131^I-MIBG therapy, which requires MIBG-avid disease.

Quantitative systems such as the Curie and SIOPEN scores can be applied at any point during therapy to assess disease burden and treatment response. A ≥ 50% reduction in score typically reflects a partial response [60], although emerging evidence indicates that the absolute post-treatment score is a stronger predictor of outcome than the percentage change. The 2017 INRC consensus recommends integrating ^123^I-MIBG score dynamics with anatomic criteria (RECIST) to define overall response. Achieving ^123^I-MIBG negativity (score 0) at the end of therapy is associated with the highest probability of durable remission, whereas persistent or recurrent uptake often heralds treatment failure—frequently preceding the onset of clinical symptoms.

## Future perspectives

### Dosimetry guided treatment

Over recent decades, ^131^I-MIBG has established itself as a key salvage treatment for relapsed or refractory neuroblastoma, particularly in pediatric patients and, in select cases, adolescents or adults with residual or recurrent disease. Traditional fixed dosing, commonly weight-based (e.g., 444 MBq/kg), fails to account for individual differences in ^131^I-MIBG uptake and clearance, increasing the risk of undertreatment or excessive toxicity, especially myelosuppression, which frequently manifests as severe neutropenia and thrombocytopenia. This variability has driven the adoption of dosimetry-guided ^131^I-MIBG therapy, in which the administered activity is tailored to each patient’s pharmacokinetics to deliver a precise radiation dose to tumors or critical organs, while typically capping bone marrow exposure [62, 63].

Dosimetry-guided approaches aim to optimize the whole-body absorbed dose (WBD) per treatment cycle to achieve effective tumor targeting with manageable marrow toxicity, with an established maximum WBD of 4 Gy. Previous studies have shown that doses above 3 Gy, when combined with chemotherapy, increased toxicity; therefore, reducing the chemotherapy dose was recommended.

Because WBD serves as a surrogate for bone marrow dose, it enables safe dose escalation, particularly when paired with autologous stem-cell rescue [62]. Two main strategies are used: prospective dosing, in which a tracer dose (e.g., ^131^I-MIBG or ^124^I-MIBG) is administered to measure kinetics before therapy, and adaptive dosing, which adjusts the absorbed dose in real time based on measurements obtained during treatment.

In a prospective dosing study, Maric et al. (2023) used ^124^I-MIBG PET imaging to guide high-dose ^131^I-MIBG therapy in 14 patients, including those with neuroblastoma [64]. Individual pharmacokinetics informed activity administration ranging from 3.5 to 50 GBq (median ~ 14 GBq), achieving therapeutic tumor doses while maintaining whole-body exposure below ~ 2 Gy. This strategy produced durable responses, with a median overall survival of ~ 85 months and minimal toxicity—only one case of self-limited grade 3 marrow suppression, and no need for stem-cell rescue. Likewise, Seo et al. (2019) at UCSF showed that ^124^I-MIBG PET dosimetry could accurately predict tumor doses and optimize treatment planning, improving efficacy while lowering risk [65].

The adaptive approach begins with a conservative initial dose, followed by post-therapy imaging to measure absorbed radiation, enabling dose adjustment in subsequent cycles. Rubio et al. (2020) compared whole-body–dose–guided therapy in five children with fixed dosing in 24 others, aiming for 4 Gy total-body absorption over two infusions [37]. Dosimetry-guided patients achieved doses within ~ 4.4% of the target, versus ± 23% with fixed dosing, minimizing under- or overdosing. Clinically, this group showed a trend toward higher 2-year event-free survival (40% vs. 16.7%), although the cohort size was limited.

Studies from the late 2010s, such as George et al. (2016), further underscored the advantages of individualized dosing. In a cohort of 25 children with refractory or relapsed neuroblastoma, ^131^I-MIBG activities were tailored according to prior treatments, disease burden, and dosimetry, aiming for ~ 2 Gy per infusion. This approach yielded a 58% overall response rate (2 CR, 13 PR), exceeding the typical 30%–45% range. Hematological toxicity, mainly transient neutropenia, was proportional to the whole-body dose, whereas no significant hepatic, renal, or thyroid injury occurred, even with cumulative activities above 30 GBq. Tumor-absorbed doses varied widely (18–81 Gy; mean ~ 44 Gy), reflecting heterogeneity in tumor features such as vascularity and norepinephrine transporter expression, reinforcing the need for patient-specific dosing to achieve optimal effect [62].

Dosimetry-guided ^131^I-MIBG therapy provides a more precise and effective strategy for neuroblastoma, delivering higher tumor radiation while limiting toxicity. By incorporating individual pharmacokinetics, it offers superior outcomes compared with fixed dosing, with evidence of improved response rates and survival. Challenges such as inter-tumor heterogeneity remain, but advances like PET-based dosimetry hold strong potential for enhancing ^131^I-MIBG’s role in the treatment of high-risk neuroblastoma.

### Emerging insights and research

Despite advances in treatment, neuroblastoma continues to pose major challenges, particularly in high-risk and relapsed/refractory cases. Nuclear medicine provides targeted therapeutic strategies, and the outlook for this field is promising, with ongoing efforts focused on developing and integrating novel radiopharmaceuticals.

One emerging direction is alpha-particle therapy. Although ^131^I-MIBG has demonstrated clinical activity, its beta-particle emissions have a relatively long path length, which may limit effectiveness against micrometastases in the bone marrow [66]. In contrast, alpha emitters such as Astatine-211 (^211^At) offer a higher linear energy transfer (LET) and a much shorter path length, making them well-suited for eradicating microscopic residual disease [66, 67]. Preclinical work with ^211^At-meta-astatobenzylguanidine (^211^At-MABG) has shown potent antitumor activity in neuroblastoma models [66]. Another promising candidate, ^211^At-MM4, targets PARP-1—an enzyme found to be overexpressed in certain neuroblastomas [68]—and has demonstrated strong efficacy in both in vitro and in vivo studies. Its cytotoxicity stems from alpha-particle-induced DNA damage rather than solely from PARP inhibition. Interestingly, fractionated dosing of ^211^At-MM4 may further enhance tumor targeting by upregulating PARP-1 expression.

Peptide receptor radionuclide therapy (PRRT) using ^177^Lu-DOTATATE (Lutathera^®^), which targets somatostatin receptor 2 (SSTR2), represents another promising avenue. SSTR2 is expressed in a large proportion of neuroblastoma tumors [69, 70]. Early phase clinical trials have shown promising results with ^177^Lu-DOTATATE in neuroblastoma treatment [69, 71]. Studies have also explored the potential of ⁶⁸Ga-DOTATATE PET/CT for imaging SSTR expression to identify patients suitable for PRRT [70, 72, 73]. Preclinical studies suggest that combining ^177^Lu-DOTATATE with other agents—such as the p53 stabilizer VIP116—can produce synergistic effects, resulting in prolonged survival and higher complete remission rates [69]. ⁶⁴/⁶⁷Cu-SARTATE, another SSTR2-targeting agent, is being investigated as a theranostic pair for the detection and treatment of minimal residual disease [74].

Radioimmunotherapy (RIT) targeting GD2—a ganglioside abundantly expressed on neuroblastoma cells—is also an active area of research [75, 76]. One approach involves ^177^Lu-labeled humanized anti-GD2 monoclonal antibody (hu3F8), which has shown selective tumor uptake and significant tumor growth inhibition in preclinical models [76]. In parallel, novel pre-targeted RIT strategies are being developed to enhance therapeutic efficacy while minimizing off-target toxicity [77, 78].

## Conclusion

In conclusion, ^131^I-MIBG remains a pivotal therapy in neuroblastoma management, providing a safe and effective option for both newly diagnosed and relapsed or refractory disease. Its future potential, however, reaches well beyond current use. Ongoing advances in nuclear medicine—particularly in refining combination strategies and developing next-generation radiopharmaceuticals—will be key to fully realizing ^131^I-MIBG’s therapeutic capabilities and driving further improvements in outcomes for patients with neuroblastoma.

## Abbreviations

NB: neuroblastoma
ORR: objective response rate (CR + PR)
CR: complete response
PR: partial response
SD: stable disease
PD: progressive disease
OS: overall survival
UKCCSG: United Kingdom Children’s Cancer Study Group

## Declarations

### **Generative AI in Scientific Writing**

During the preparation of this work, the authors used Grammarly to improve the language and readability. After using this service, the authors reviewed and revised the content as necessary and take full responsibility for the final content of the publication.
